# Association between host species choice and morphological characters of main sensory structures of * Culicoides* in the Palaeartic region

**DOI:** 10.7717/peerj.3478

**Published:** 2017-07-27

**Authors:** Denis Augot, Leila Hadj-Henni, Stavana E. Strutz, Darine Slama, Christine Millot, Jérôme Depaquit, Jean-Marc Millot

**Affiliations:** 1 Usc VECPAR, AE 4688, UFR Cap Sante, Université Champagne-Ardenne, UFR Pharmacie, ANSES, Reims, France; 2 University of Texas at Austin, United States of America; 3 Laboratoire de Parasitologie-Mycologie, 99UR/08-05, Département de biologie clinique, Faculté de Pharmacie de Monastir, Monastir, Tunisia; 4 Laboratory of Parasitology-Mycology, National Reference Centre for Toxoplasmosis, Biological Resource Centre Toxoplasma, Maison Blanche Hospital, Reims, France; 5 Laboratoire de Recherche en Nanosciences (LRN)-EA4682, Université de Reims Champagne-Ardenne, Reims, France

**Keywords:** *Culicoides*, Sensory structures, Host preference, Vectors

## Abstract

*Culicoides* (Diptera: Ceratopogonidae) serve as vectors of several mammalian and avian diseases, including bluetongue, Schmallenberg, African horse sickness, avian malaria and Oropouche. Host preference investigations are necessary to assess the transmission routes of vector-borne diseases and to inform mitigation strategies. A recent study examining the main sensory structures (palps and antennae) of * Culicoides* species suggests that they be classified as ornithophilic or mammalophilic according to their feeding habits. We analyzed *Culicoides* host preferences according to the literature and carried out a multiple correspondence analysis linking these preferences with morphological data. Seven out of 12 variables were found to be reliable predictors of host preference in *Culicoides* species: Antenna Flagellomer-Sensilla Coeloconica-Number: (7–10) and (11–13); Antenna Flagellomer-Sensilla Coeloconica IV–X: presence; Palpus-size: wide and/or narrow opening and shallow pit; Palpus-Shape: strongly swollen; Antenna-Short sensilla trichodea-distal part segment IV to X-Number: 2 seta each. Our results demonstrate that the presence of *sensilla coeloconica* and the maxillary palpus can be used to separate ornithophilic and mammalophilic or ornithophilic/mammalophilic species.

## Introduction

Vector-borne diseases are health problems for humans, livestock, and wild animals and are transmitted by a variety of arthropods. *Culicoides* species constitute a diverse and widespread genus with more than 1,400 species world-wide (Borkent, 2014). Biting midges transmit multiple avian and mammalian diseases, including Bluetongue virus (BTV), Schmallenberg virus (SV), epizootic haemorrhagic disease virus (EHDV), African horse sickness virus (AHSV) and avian *Haemoproteus* (subgenus *Parahaemoproteus*) parasites.

Knowledge of host preferences and feeding behavior are essential to understanding pathogen transmission cycles and the epidemiology of their associated diseases. Host preferences of *Culicoides* have been investigated using two laboratory methods: (i) serological analysis of visible abdominal blood via the precipitin test (Braverman, Boreham & Galum, 1971; Walker & Davies, 1971; Nevill & Anderson, 1972) or ELISA test (Blackwell, Mordue , Luntz; Blackwell, Brown & Mordue, 1995); (ii) polymerase chain reaction (PCR) using several genes (Bartsch et al., 2009; Votypka, Synek & Svobodova, 2009; Garros et al., 2011; Lassen et al., 2011; Lassen, Nielsen & Kristensen, 2012; Ninio et al., 2011; Calvo et al., 2012; Martínez-de la Puente et al., 2012; Pettersson et al., 2013; Bobeva et al., 2015; Hadj-Henni et al., 2015; Slama et al., 2015). Observation based studies have also been used to assess host preference: adult *Culicoides* females have been directly collected from bait animals with sticky traps, by aspiration on bait animals, and with light or animal-baited traps (Viennet et al., 2011; Braverman et al., 2012; Ayllón et al., 2014; Elbers & Meiswinkel, 2014; Thompson et al., 2014; Elbers & Meiswinkel, 2015).

Direct collection from animals has been considered the most reliable method to study the vector/host ratio (Silver & Service, 2008), which is an essential parameter for the modeling of vectorial capacity and virus transmission (Garrett-Jones, 1964). Various factors, such as habitat type, season, and bait species, contribute to the capture success of engorged females when using light traps. Engorged biting midges can be either fully engorged or have partially digested blood meals. Only fully engorged females were considered for blood meal identification; and the percentage of the engorged females using UV traps was low (Martínez-de la Puente, Figuerola &Soriguer, 2015). The percentage varied from 0.97% to 27.7% with three studies presenting a percentage of engorged females greater than 10% and seven studies presenting a percentage of less than 5% (Bartsch et al., 2009; Votypka, Synek & Svobodova, 2009; Lassen et al., 2011; Lassen, Nielsen & Kristensen, 2012; Garros et al., 2011; Ninio et al., 2011; Martínez-de la Puente et al., 2012; Santiago-Alarcon et al., 2012; Pettersson et al., 2013; Slama et al., 2015; Hadj-Henni et al., 2015; Bobeva et al., 2015).

*Culicoides* species are mainly mammalophilic and/or ornithophilic but females have also been found to occasionally feed on engorged insects (Ma et al., 2013). Some species of *Forcipomyia* and *Leptoconops* (Ceratopogonidae) feed on reptiles and frogs (Borkent, 2005). Hematophagous insects have highly developed olfactory systems and mainly use their antennae and, in some cases, maxillary palps, to detect semiochemicals. Semiochemicals can provide information about the location, suitability, or physiological state of conspecifics, hosts, or breeding sites (Logan & Birkett, 2007). Moreover, several studies carried out on feeding patterns of biting midges found variation in host attractiveness to be correlated with exhaled carbon dioxide (CO_2_), 1-octen-3-ol, lactic acid, acetone (Zimmer et al., 2015), specific phenolic compounds emitted from urine, (Bhasin, Luntz & Mordue, 2001) or hair fragrance (Mands, Kline & Blackwell, 2004).

Consequently, the morphological characterization of the *Culicoides* sensory structures can serve as an indirect method to assess host preference (Jamnback, 1965; Braverman & Hulley, 1979; McKeever, Hagan & Wang, 1994; Blackwell, Mordue & Mordue, 1994). Here, we investigate how differences in morphological characters of the sensory structures of female *Culicoides* may impact host feeding choice. Specific objectives included morphological analysis of the main sensory structures previously explored by Blackwell (2004), Braverman et al. (2012) and Talavera et al. (2015) using host species published in the literature (engorged females and animal baits). We analyzed whether a combination of morphological variables could be used to predict host preference.

## Materials & Methods

Table 1 summarizes host species of biting females of species of *Culicoides* identified using molecular methods and animal baits. In order to exclude variability of morphological characters, we use an Interactive Identification Key for *Culicoides* (Mathieu et al., 2012). The raw dataset included 12 morphological characters (Table 2): (1) Antenna Flagellomer-Sensilla coeloconica- number with [0]: 0–6, [1]: 7–10, [2]: 11–13; (2) Antenna Flagellomer-Sensilla coeloconica-Segment-IV-X with [0]: absence, [1]: presence; (3) Antenna Flagellomer-Sensilla coeloconica-Segment-XI-XV (H16) with [0]: absence, [1]: presence; (4) Antenna-Short-segment-Shape-Flask-Shape (H09) with [0]: inflated, [1]: flask shape, [2]: inflated and flask; (5) Antenna-Short sensilla trichodea, distal part segment IV to X-Number (H11) with [0]: 2 seta each, [1]: 1 seta each; (6) Antenna segment XI/X ratio, length of segment XI divided by length of segment X (H13); (7) Palp-3rd palpal segment-sensory pits-Number (H07) with [0]: multiple, [1]: single, [2]: multiple and single; (8) Palp-3rd palpal segment-single sensory pit-opening versus depth = large/small ; (H08) with[0]:small, [1]: wide opening and shallow pit, [2]: narrow opening and shallow pit, wide opening and shallow pit; (9) Palp-3rd palpal segment-Shape (H06) with [0]: strongly swollen, [1]: triangular and moderately swollen, [2]: slender or slightly swollen, triangular and moderately swollen, [3]: lender or slightly swollen; (10) Cibarial-Armature (H04); (11) Pharynx posterior-Armature- (H05); (12) Eyes-Inter Ocular-Space-Shape (H02). For the size of the maxillary palpus, only a single sensory pit was used. However, if a *Culicoides* specimen had multiple irregular pits then we classified them as a small opening. All specimens in this study present a *sensilla coeloconica* in segment III. Therefore, a new group (Segment-IV-X) has been drawn according to Talavera et al. (2015). Finally, *Culicoides* species were classified into ornithophilic (O) and mammalophilic (M) or ornithophilic/mammalophilic (O, M) according to their host species (Table 1).

**Table 1:** Host preference of *Culicoides* species, based on animal baits and molecular analysis of engorged *Culicoides* females.

Species	Host preference			References
	Host	Mammalophilic (**M**) or ornithophilic (**O**) species	Primers used for bloodmeals identifcation	
*C. achrayi*	*Equus caballus, Homo sapiens, Equus asinus, Gallus gallus*	**M, O**	PNOC, Cytb	Hadj-Henni et al. (2015)
	*Bos taurus*		PNOC, COI-Cyt^d^	Ninio et al. (2011), Pettersson et al. (2013)
	Cattle, sheep, Shetland pony			Viennet et al. (2011), Ayllón et al. (2014), Elbers & Meiswinkel (2014), Elbers & Meiswinkel (2015)
*C. alazanicus*	*Anthus trivialis, Ardea purpurea , Asio otus, Columba palumbus, Delichon urbica, Ixobrychus minutus, Luscinia luscinia, Muscicapa striata, Oriolus oriolus, Parus major, Phylloscopus trochilus, Pica pica, Sylvia borin, Turdus merula, Turdus philomelos, Homo sapiens*	**M, O**	Cytb	Bobeva et al. (2014) & Bobeva et al. (2015)
*C. albicans*	Cow	**M**		Elbers & Meiswinkel (2014)
*C. brunnicans*	*Ovis aries*	**M**	Cytb^a^	Garros et al. (2011)
	*Bos taurus*		PNOC	Ninio et al. (2011)
	*Equus caballus*		PNOC, Cytb	Hadj-Henni et al. (2015)
	*Sheep*			Viennet et al. (2011)
*C. cataneii*	*Mus musculus*	**M**	PNOC, Cytb	Slama et al. (2015)
	cattle, man			Braverman et al. (2012)
*C. chiopterus*	*Bos taurus*	**M, O**	PNOC, Cytb, Cytb^a^, Cytb^c^	Lassen et al. (2011), Lassen, Nielsen & Kristensen (2012), Garros et al. (2011), Ninio et al. (2011), Hadj-Henni et al. (2015)
	*Columba palumbus*		COI-Cytb^b^	Lassen et al. (2011)
	*Ovis aries*		Cytb^a^	Garros et al. (2011)
	*Capra hircus*		Cytb^a^	Garros et al. (2011)
	*Homo sapiens*		COI	Santiago-Alarcon et al. (2012)
	*Equus caballus*		PNOC, Cytb, COI-Cyt^d^	Pettersson et al. (2013), Hadj-Henni et al. (2015)
	*Capreolus capreolus*		Cytb^c^	Lassen, Nielsen & Kristensen (2012)
	Cow, sheep, Shetland pony			Viennet et al. (2011), Elbers & Meiswinkel (2014), Elbers & Meiswinkel (2015), Ayllón et al. (2014), Santiago-Alarcon et al. (2012)
	Dog			Mezenev (1990)
*C. circumscriptus*	Birds, cattle, man, rabbit, sheep	**M, O**		Viennet et al. (2011), Ferraguti et al. (2013); in Braverman et al. (2012)
	*Asio otus*		Cytb	Bobeva et al. (2014), Bobeva et al. (2015)
	*Homo sapiens*		PNOC, Cytb, Cytb^c^	Lassen, Nielsen & Kristensen (2012), Slama et al. (2015)
	*Phylloscopus trochilus, Corvus corone, Turdus philomelos, Pica pica, Columba palumbus*		COI-Cyt^d^	Pettersson et al. (2013)
	*Pica pica, Turdus merula*		Cytb^c^	Lassen, Nielsen & Kristensen (2012), Bobeva et al. (2014)
*C. clastrieri*	*Homo sapiens*	**M, O**	COI	Santiago-Alarcon et al. (2012)
	*Birds (Tadorna ferruginea, Turdus philomelos)*			Santiago-Alarcon et al. (2013)
*C. deltus*	Horses, cows, man	**M**		Santiago-Alarcon et al. (2013)
	*Homo sap iens*		COI	Santiago-Alarcon et al. (2012)
	*Bos taurus*		COI-Cytb^b^	Lassen et al. (2011)
*C. dewulfi*	Cattle, Sheep, Shetland pony	**M**		Elbers & Meiswinkel (2014), Elbers & Meiswinkel (2015), Ayllón et al. (2014), Viennet et al. (2011)
	*Homo sapiens*		Cytb^c^, COI	Santiago-Alarcon et al. (2012), Lassen, Nielsen & Kristensen (2012)
	*Bos taurus*		PNOC, Cytb^a^, Cytb^c^	Garros et al. (2011), Ninio et al. (2011), Lassen, Nielsen & Kristensen (2012)
	*Ovis aries*		Cytb^a^	Garros et al. (2011)
	*Equus caballus*		PNOC, COI-Cyt^d^	Ninio et al. (2011), Pettersson et al. (2013)
	*Oryctolagus cuniculus, Sus scrofa*		PNOC	Ninio et al. (2011)
*C. duddingstoni*	*Passer montanus, Cyanistes caeruleus, Pica pica, Passer domesticus*	**O**	COI-Cyt^d^	Pettersson et al. (2013)
	*Garrulus glandarius*		Cytb^c^	Lassen, Nielsen & Kristensen (2012)
*C. fagineus*	cattle, man	**M**		Braverman et al. (2012)
*C. fascipennis*	cattle, man, birds, rabbit, Shetland pony	**M, O**		Elbers & Meiswinkel (2014), Elbers & Meiswinkel (2015), Braverman et al. (2012)
	Dog			Mezenev (1990)
*C. festivipennis*	*Ovis aries*	**M, O**	Cytb	Calvo et al. (2012)
	*Homo sapiens*		COI, Cytb	Calvo et al. (2012), Santiago-Alarcon et al. (2012),
	*Pica pica, Tur dus philomelos*		COI-Cyt^d^	Pettersson et al. (2013)
	*Anthus trivialis, Asio otus, Nycticorax nycticorax, Oriolus oriolus, Passer domesticus, Passer hispaniolensis, Passer montanus, Pica pica, Streptopelia decaocto*		Cytb	Bobeva et al. (2014), Bobeva et al. (2015)
	*Homo sapiens*			Santiago-Alarcon et al. (2012), Santiago-Alarcon et al. (2013)
	*Columba palumbus*		Cytb^c^	Lassen, Nielsen & Kristensen (2012)
	poultry, birds, man, cows			Santiago-Alarcon et al. (2012), Santiago-Alarcon et al. (2013), Braverman et al. (2012), Elbers & Meiswinkel (2015)
*C. furcillatus*	*Oryctolagus cuniculus*	**M**	PNOC	Ninio et al. (2011)
	*Bos taurus*		Cytb^c^	Lassen, Nielsen & Kristensen (2012)
	*Equus caballus, Homo sapiens*		PNOC, Cytb	Hadj-Henni et al. (2015)
*C gejgelensis*	cattle, man	**M**		in Braverman et al. (2012)
*C. griseidorsum*	cattle, sheep, horses, donkeys	**M, O**		Ayllón et al. (2014), Braverman et al. (2012)
	*Coccothraustes coccothraustes, Luscinia megarhynchos, Pica pica, Cervus elaphus*			Bobeva et al. (2015)
*C. grisescens*	Cattle	**M**		Elbers & Meiswinkel (2015)
	*Bos Taurus*		COI-Cyt^d^	Pettersson et al. (2013)
*C. haranti*	cattle, man	**M**		Braverman et al. (2012)
*C. heliophilus*	man, cows, sheep, dogs, Shetland pony	**M**		Elbers & Meiswinkel (2014), Elbers & Meiswinkel (2015), Santiago-Alarcon et al. (2013)
*C. imicola*	Horse, sheep, cattle, ibex, pig, poultry	**M, O**		Fall et al. (2015), Braverman et al. (2012)
	*Homo sapiens, Capra hircus, Ovis aries, Canis lupus familiaris, Lanius meridonalis*		PNOC, Cytb	Slama et al. (2015)
*C. impunctatus*	Birds, cow, ewe, Shetland pony	**M, O**		Ziegyte et al. (2014), Elbers & Meiswinkel (2014), Elbers & Meiswinkel (2015), Santiago-Alarcon et al. (2013)
	*Equus caballus, Ovis aries*		COI-Cyt^d^	Pettersson et al. (2013)
*C. jumineri*	*Homo sapiens, Bos taurus, Mustela nivalis, Gallus gallus, Drosophila melanogaster, Carlia fusca, Aedes sp.*	**M, O**	PNOC, Cytb	Slama et al. (2015)
*C. kibunensis*	cattle, man, birds	**M, O**		Santiago-Alarcon et al. (2013), Braverman et al. (2012)
	*Bos taurus*		Cytb^c^	Lassen, Nielsen & Kristensen (2012)
	*Homo sapiens, Sylvia atricapilla*		COI	Santiago-Alarcon et al. (2012), Santiago-Alarcon et al. (2013)
	*Acrocephalus pa lustris, Columba palumbus, Emberiza citrinella*		Cytb^c^	Lassen, Nielsen & Kristensen (2012)
	Erithacus rubecula			Santiago-Alarcon et al. (2013)
*C. longipennis*	cattle, man	**M**		Braverman et al. (2012)
*C. lupicaris*	Cow, sheep	**M**		Viennet et al. (2011), Ayllón et al. (2014), Elbers & Meiswinkel (2014), Elbers & Meiswinkel (2015)
	*Ovis aries*		Cytb^a^	Garros et al. (2011)
	*Equus caballus*		PNOC, Cytb	Ninio et al. (2011), Hadj-Henni et al. (2015)
	*Homo sapie ns, Equus asinus*		PNOC, Cytb	Hadj-Henni et al. (2015)
	*Oryctolagus cuniculus, Bos taurus, Sus scrofa*		PNOC	Ninio et al. (2011)
*C. maritimus*	cattle, man, rabbit	**M**		Braverman et al. (2012)
*C. minutissimus*	*Pica pica*	**O**	Cytb	Votypka, Synek & Svobodova (2009)
*C. montanus*	cattle, man	**M**		Braverman et al. (2012)
*C. newsteadi*	cattle, horse, poultry, man	**M, O**		Santiago-Alarcon et al. (2013), Braverman et al. (2012)
	*Ovis aries*		PNOC, Cytb, Cytb^a^	Garros et al. (2011), Calvo et al. (2012), Slama et al. (2015)
	*Bos taurus*		COI-Cyt^d^	Pettersson et al. (2013)
	*Homo sapiens*		PNOC, Cytb	Slama et al. (2015), Hadj-Henni et al. (2015)
	*Capra hircus, Meleagris gallopavo, Gallus gallus*		PNOC, Cytb	Slama et al. (2015)
	*Equus caballus, Equus asinus*		PNOC, Cytb	Hadj-Henni et al. (2015)
*C. obsoletus*	Sheep, horse, man, cattle, bird, poultry, livestock, Shetland pony	**M, O**		Viennet et al. (2011), Santiago-Alarcon et al. (2013), Braverman et al. (2012), Elbers & Meiswinkel (2015)
	*Bos taurus*		PNOC, Cytb^a^, COI-Cytb^b^, Cytb^c^, COI, COI-Cyt^d^	Lassen et al. (2011), Lassen, Nielsen & Kristensen (2012), Garros et al. (2011), Ninio et al. (2011), Calvo et al. (2012), Santiago-Alarcon et al. (2012), Pettersson et al. (2013), Hadj-Henni et al. (2015)
	*Equus caballus*		PNOC, COI-Cytb^b^, Cytb^c^, COI-Cyt^d^	Lassen et al. (2011), Lassen, Nielsen & Kristensen (2012), Ninio et al. (2011), Pettersson et al. (2013), Hadj-Henni et al. (2015)
	*Anas platyrhynchos, Columba palumbus*		COI-Cytb^b^	Lassen et al. (2011)
	*Ovis aries*		PNOC, Cytb^a^, Cytb^c^, COI-Cyt^d^	Garros et al. (2011), Ninio et al. (2011), Calvo et al. (2012), Lassen, Nielsen & Kristensen (2012), Pettersson et al. (2013)
	*Oryctolagus cuniculus*		PNOC	Ninio et al. (2011)
	*Homo sapiens*		PNOC, Cytb, Cytb^c^, COI	Ninio et al. (2011), Calvo et al. (2012), Lassen, Nielsen & Kristensen (2012), Santiago-Alarcon et al. (2012), Santiago-Alarcon et al. (2013), Hadj-Henni et al. (2015)
	*Gallus gallus, Microtus savii*		Cytb	Calvo et al. (2012)
	*Capreolus capreolus, Capra hircus, Cervus elaphus, Mus musculus*		Cytb^c^	Lassen, Nielsen & Kristensen (2012)
	*Equus asinus, Felis silvestris*		PNOC, Cytb	Hadj-Henni et al. (2015)
*C. pallidicornis*	Sheep, cows, birds, man, Shetland pony	**M, O**		Santiago-Alarcon et al. (2012), Santiago-Alarcon et al. (2013), Ayllón et al. (2014), Elbers & Meiswinkel (2014), Elbers & Meiswinkel (2015)
	*Oryctolagus cuniculus, Bos taurus*		PNOC	Ninio et al. (2011)
	*Capra hircus*		Cytb^c^	Lassen, Nielsen & Kristensen (2012)
	*Homo sapiens*		COI	Santiago-Alarcon et al. (2012)
*C. parroti*	*Ovis aries*	**M**	Cytb	Calvo et al. (2012)
*C. pictipennis*	Cow, birds, man	**M, O**		Elbers & Meiswinkel (2014), Santiago-Alarcon et al. (2012)
	*Pica pica*		Cytb, Cytb^c^	Votypka, Synek & Svobodova (2009), Lassen, Nielsen & Kristensen (2012)
	*Ovis aries*		Cytb^a^	Garros et al. (2011)
	*Turdus merula, H omo sapiens*		COI	Santiago-Alarcon et al. (2012)
	*Parus major*		COI-Cyt^d^	Pettersson et al. (2013)
	*Erithacus rubecula*			Santiago-Alarcon et al. (2013)
	*Bos taurus, Cervus elaphus*			Bobeva et al. (2015)
*C. picturatus*	Sheep	**M**		Viennet et al. (2011)
	*Bos taurus*		PNOC	Ninio et al. (2011)
*C. poperinghensis*	*Man*	**M**		Santiago-Alarcon et al. (2013)
			*Bos taurus*		PNOC, Cytb^c^	Ninio et al. (2011), Lassen, Nielsen & Kristensen (2012)
	*Homo sapiens*		COI	Santiago-Alarcon et al. (2012)
*C. pulicaris*	Cows, sheep, horses, buffaloes, man, cattle, Shetland pony	**M, O**		Viennet et al. (2011), Santiago-Alarcon et al. (2012), Santiago-Alarcon et al. (2013), Ayllón et al. (2014), Elbers & Meiswinkel (2014), Elbers & Meiswinkel (2015), Braverman et al. (2012)
	Dog			Mezenev (1990)
	*Bos taurus*		PNOC, Cytb, COI-Cytb^b^, Cytb^c^	Lassen et al. (2011), Lassen, Nielsen & Kristensen (2012), Calvo et al. (2012), Hadj-Henni et al. (2015)
	*Oryctolagus cuniculus*		PNOC	Ninio et al. (2011)
	*Ovis aries, Gallus gallus*		Cytb	Calvo et al. (2012)
	*Homo sapiens*		PNOC, Cytb, COI	Calvo et al. (2012), Santiago-Alarcon et al. (2012), Hadj-Henni et al. (2015)
	*Equus caballus*		PNOC, Cytb	Lassen, Nielsen & Kristensen (2012), Hadj-Henni et al. (2015)
	*Capra hircus, Cervus elaphus*		Cytb^c^	Lassen, Nielsen & Kristensen (2012)
*C. punctatus*	cattle, man, birds, rabbit, sheep, Shetland pony	**M, O**		Viennet et al. (2011), Ayllón et al. (2014), Elbers & Meiswinkel (2014), Elbers & Meiswinkel (2015), Braverman et al. (2012), Santiago-Alarcon et al. (2012), Santiago-Alarcon et al. (2013)
	*Bos taurus*		PNOC, Cytb^a^, COI-Cytb^b^, Cytb^c^, COI-Cyt^d^	Lassen et al. (2011), Lassen, Nielsen & Kristensen (2012), Garros et al. (2011), Ninio et al. (2011), Pettersson et al. (2013)
	*Capra hircus, Capreolus capreolus*		Cytb^c^	Lassen, Nielsen & Kristensen (2012)
	*Equus caballus*		PNOC, Cytb, COI-Cytb^b^, COI-Cyt^d^	Lassen et al. (2011), Ninio et al. (2011), Pettersson et al. (2013), Hadj-Henni et al. (2015)
	*Anas platyrhynchos, Columba palumbus*		COI-Cytb^b^	Lassen et al. (2011)
	*Oryctolagus cuniculus*		PNOC	Ninio et al. (2011)
	*Ovis aries*		COI-Cyt^d^	Calvo et al. (2012); Pettersson et al. (2013)
	*Homo sapiens*		PNOC, Cytb	Calvo et al. (2012), Bobeva et al. (2014), Hadj-Henni et al. (2015)
	*Gallus gallus, Microtus savii*		Cytb	Calvo et al. (2012)
	*Alces alces, Luscinia svecica*		COI-Cyt^d^	Pettersson et al. (2013)
	*Equus asinus*		PNOC, Cytb	Hadj-Henni et al. (2015)
	*Cervus elaphus*			Bobeva et al. (2015)
*C. puncticollis*	cattle, horses, man; donkey	**M**		Braverman et al. (2012)
*C. reconditus*	*Pica pica*	**O**	Cytb^c^	Lassen, Nielsen & Kristensen (2012)
*C. riethi*	Shetland pony	**M**		Elbers & Meiswinkel (2015)
	*Bos taurus*		Cytb^c^	Lassen, Nielsen & Kristensen (2012)
*C. salinarius*	*Columba palumbus*	**O**	COI-Cyt^d^	Pettersson et al. (2013)
	*Passer montanus*		Cytb^c^	Lassen, Nielsen & Kristensen (2012)
*C. santonicus*	*Sheep*	**M**		Viennet et al. (2011)
*C. scoticus*	Sheep , cows, horses, Shetland pony, man	**M, O**		Viennet et al. (2011), Santiago-Alarcon et al. (2012), Santiago-Alarcon et al. (2013), Elbers & Meiswinkel (2015)
	*Bos taurus*		PNOC, COI-Cytb^b^, Cytb^c^, COI-Cyt^d^	Lassen et al. (2011), Lassen, Nielsen & Kristensen (2012), Ninio et al. (2011), Pettersson et al. (2013)
	*Capreolus capreolus*		COI-Cytb^b^, Cytb^c^	Lassen et al. (2011), Lassen, Nielsen & Kristensen (2012)
	*Anas platyrhynchos, Columba palumbus*		COI-Cytb^b^	Lassen et al. (2011)
	*Equus caballus*		PNOC, Cytb, Cytb^c^, COI, COI-Cyt^d^	Ninio et al. (2011), Lassen, Nielsen & Kristensen (2012), Santiago-Alarcon et al. (2012), Pettersson et al. (2013), Hadj-Henni et al. (2015)
	*Ovis aries*		PNOC, COI-Cyt^d^	Ninio et al. (2011), Pettersson et al. (2013)
	*Oryctolagus cuniculus, Sus scrofa*		PNOC	Ninio et al. (2011)
	*Homo sapiens*		PNOC, Cytb, Cytb^c^, COI	Lassen, Nielsen & Kristensen (2012), Santiago-Alarcon et al. (2012), Hadj-Henni et al. (2015)
*C. segnis*	*Cow, sheep*	**M**		Elbers & Meiswinkel (2015)
*C. semimaculatus*	cattle, man	**M, O**	COI	Braverman et al. (2012)
	*Homo sapiens*			Santiago-Alarcon et al. (2012)
	*Birds* (Erithacus rubecula)			Santiago-Alarcon et al. (2013)
*C. shaklawensis*	cattle, man, sheep	**M**		Viennet et al. (2011), Braverman et al. (2012)
*C. simulator*	Sheep	**M**		Viennet et al. (2011)
*C. stigma*	Cow, sheep, man, Shetland pony	**M**		Elbers & Meiswinkel (2014), Elbers & Meiswinkel (2015), Ayllón et al. (2014), Santiago-Alarcon et al. (2012), Santiago-Alarcon et al. (2013)
*C. subfagineus*	Cattle	**M**		Ayllón et al. (2014)
*C. subfasciipennis*	Cow, sheep	**M**		Viennet et al. (2011), Ayllón et al. (2014), Elbers & Meiswinkel (2014), Elbers & Meiswinkel (2015)
	*Equus caballus, Homo sapiens*		PNOC, Cytb	Hadj-Henni et al. (2015)
*C. truncorum*	*Pica pica*	**O**	Cytb	Votypka, Synek & Svobodova (2009)
*C. vexans*	Sheep, horses, goats, man, birds, cow	**M, O**		Viennet et al. (2011), Santiago-Alarcon et al. (2012), Santiago-Alarcon et al. (2013), Elbers & Meiswinkel (2015)
	*Homo sapiens*		PNOC, Cytb, Cytb^c^	Lassen, Nielsen & Kristensen (2012), Hadj-Henni et al. (2015)
	*Bos taurus, Capreolus capreolus*		Cytb^c^	Lassen, Nielsen & Kristensen (2012)

**Notes.**

Cytb^a^ specific multiplex PCR based on Cytb polymorphism COI-Cytb^b^ five primer pairs amplifying different regions of mtDNA(COI or Cytb): Mammal, Avian, COI short, Cytb, Cow121F Cytb^c^ specific cytb primer par for cow and universal cytochrome b primer COI-Cyt^d^ Cytb, COI, Sheep universal, Sheep –specific, Human

For the statistical analysis, the morphological characteristics and species classification are coded as qualitative variables (see Data S1). Therefore, quantitative methods such as the Principal Coordinates Analysis (PCO) or Nonmetric Multidimensional Scaling (NMDS) are not applicable here. Multiple correspondence analysis (MCA) is a data analysis technique for qualitative variables (Greenacre & Jörg, 2006), to obtain maps showing the distances between the qualitative variables and the observations. MCA was performed with XLSTAT software and used to explore the correlation structure between morphological characteristics and host preference. Within the indicator matrix, the rows represented individuals and the columns represented categories of the variables. Correspondence analysis was applied through the symmetric matrix of all two-way cross-tabulations, to present the indicator matrix in a low-dimensional Euclidean space. The first axis was found to be the most important dimension, the second axis the second most important, and so on, in terms of the amount of variance accounted for. All *Culicoides* species (Data S1) were first coded into a 12, 10 or 7 morphological characteristics indicator matrix and analyzed by MCA to obtain the discriminant factors. All species were then projected in order to indicate the species preferences. The MCA map showed that the inertia for the two first dimensions is >70%. So, 3 groups were obtained into three ellipses, based on the F1 and F2 axis. The ellipses were built with the average of each group ± 1 SD (standard deviation), includes about 68% of the observations. A student test was used to compare the several categories (M vs. M/O and O vs. M/O).

## Results

**Figure 1:**
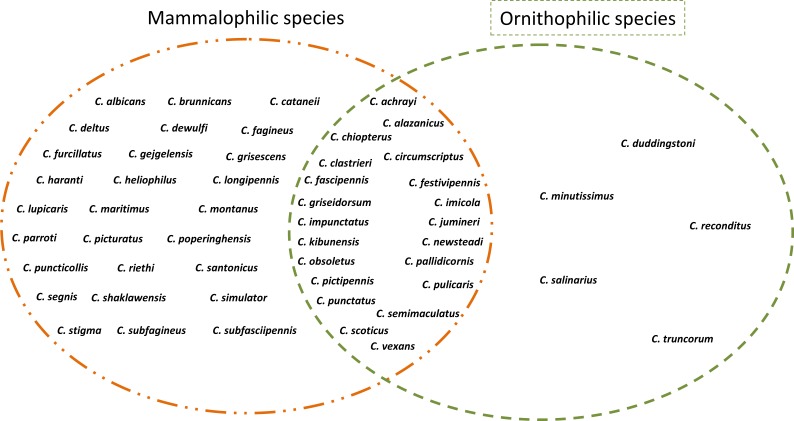
Host preference behavior of *Culicoides* in the Palaeartic region collected from the literature.

**Figure 2:**
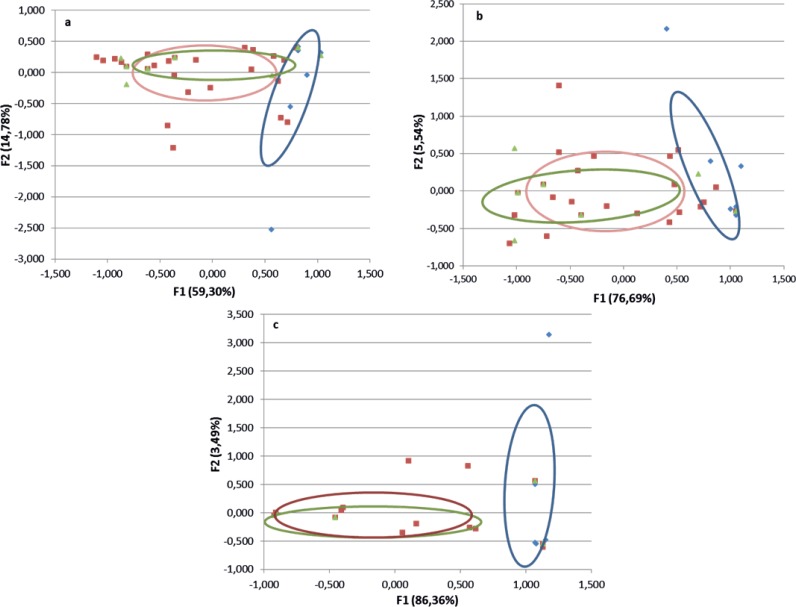
Results of multicomponent analyses of morphological characteristics of *Culicoides* and host preference. Results based on 10 characteristics (A); seven characteristics (B) and four characteristics (C) according to Talavera et al. (2015) (Red: Mammals; green: mammals and birds and blue: birds).

Overall, 53 species were investigated. Five species (*C. duddingstoni*, *C. minutissimus*, *C. reconditus*, *C. salinarius* and *C. truncorum*) are ornithophilic, 27 are mammalophilic and 21 are ornithophilic/mammalophilic (Fig. 1). Multiple correspondence analysis locates all the categories in a Euclidean space. The MCA map showed that the inertia for the two first dimensions is ranging to 74% and 90% (Fig. 2). The first dimension explained more of 59% of data variability, and the categories are mainly organized along this axis. In the Fig. 2, each point (red, green, blue) corresponds to a *Culicoides* species category and several species could be plotted in the same point.

A first MCA allowed a separation between ornithophilic (O), mammalophilic (M) and ornithophilic/mammalophilic (O/M) (results not shown but very similar to those of the Fig. 2A). A second analysis using ten items (H05 and H13 morphological characters were not discriminants) showed similar results (Fig. 2A) with 3 ellipses. Finally, clearer resolution was obtained with seven morphological characteristics Antenna Flagellomer-Sensilla coeloconica- number; Antenna Flagellomer- Sensilla coeloconica-Segment-IV-X-Presence; Antenna-Short sensilla trichodea, distal part segment IV to X-Number: 1 seta each (H11); Palp-3rd palpal segment-sensory pits-Number (H07); Palp-3rd palpal segment-single sensory pit-opening versus depth = large/small (H08); Palp-3rd palpal segment-Shape (H06); Eyes-Inter Ocular-Space-Shape (H02) than with ten characteristics (Fig. 2B). The mammals (in red), Mammals/birds (in green) and birds (blue) categories are more clustered in Fig. 2A and Fig. 2C than in Fig. 2B. The Fig. 2C presents approximately the same topology than that of Fig. 2A and Fig. 2B.

Table 2 shows a good discrimination between ornithophilic/mammalophilic species and ornithophilic (*p* < 10^−4^) with F1 axis based on 10, 7 and 4 morphological characters. The first axis, called F1, separates ornithophilic and mammalophilic or ornithophilic/mammalophilic species. In constrast, the second axis F2 doesn’t separate the species (Table 3).

**Table 2:** List of morphological characters used in our study. Results of multiple component analyses with 10, 7 and 4 morphological characteristics. The F1 axis sufficiently separated feeding preferences while the F2 axis did not. Numbers in bold were correlated with host choice.

Characters			10 characters		7 characters		4 characters	
Name	Codage		Axes					
			F1	F2	F1	F2	F1	F2
Antenna Flagellomer-Sensilla coeloconica- number	0	0–6	−0.75	0.02	−0.71	−0.12	−0.76	−0.09
	1	7–10	**0.92**	−1.11	**0.79**	1.05	**0.87**	1.80
	2	11–13	**1.00**	0.55	**1.06**	−0.31	**1.06**	−0.76
Antenna Flagellomer-Sensilla coeloconica-Segment-IV-X	0	Absence	−0.83	0.28	−0.80	−0.37	−0.85	−0.10
	1	Presence	**0.80**	−0.27	**0.77**	0.36	0.82	0.10
Antenna Flagellomer-Sensilla coeloconica-Segment-XI-XV	0	Absence	0.40	−1.93				
	1	Presence	−0.07	0.34				
Palp-3rd palpal segment-sensory pits-Number	0	Multiple	−0.71	0.23	−0.67	−0.38	−0.78	0.11
	1	Single	0.63	0.01	0.63	0.04	0.70	−0.08
	2	Multiple, single	−0.73	−1.28	−0.85	1.77	−0.81	−0.007
Palp-3rd palpal segment-single sensory pit-opening versus depth = large/small	0	small	−0.52	−0.03	−0.52	0.03	−0.57	0.09
	1	Wide opening and shallow pit	**1.01**	0.35	**1.05**	−0.31	**1.09**	−0.52
	2	Narrow opening and shallow pit, wide opening and shallow pit	**0.84**	−4.85	**0.52**	4.19	**1.44**	5,44
Palp-3rd palpal segment-Shape	0	Strongly swollen	**1.12**	−0.38	**1.07**	0.54		
	1	Triangular and moderately swollen	0.48	0.25	0.52	−0.26		
	2	Slender or slightly swollen, triangular and moderately swollen	−0.84	−0.39	−0.93	0.60		
	3	Slender or slightly swollen	−1.18	−0.03	−1.12	−0.47		
Antenna-Short-segment-Shape-Flask-Shape	0	Inflated	1.29	−0.54				
	1	Flask shape	−0.11	0.15				
	2	Inflated and flask	0.14	−2.66				
Antenna-Short sensilla trichodea, distal part segment IV to X-Number	0	2 seta each	**1.01**	0.43	**1.06**	−0.27		
	1	1 seta each	−0.56	−0.24	−0.59	0.15		
Cibarial-Armature	0	Absence	0.07	−0.01				
	1	Presence	−1.09	0.13				
Eyes-Inter Ocular-Space-Shape	1	Separated narrowly	0.62	0.25	0.64	−0.20		
	2	Joined for a short distance	−1.11	0.15	−1.12	0.006		
	3	Separated narrowly, joined for a short distance or Joined for a short distance and joined for a long distance	−1.12	0.50	−1.12	−1.22		
	4	Joined for a long distance	−1.53	0.46	−1.37	−1.35		
	5	Separated widely	−0.26	−2.36	−0.43	2.31		

Table 2 shows the results of multiple component analyses obtained with 4, 7 and 10 characteristics. Finally, seven morphological characteristics (Antenna Flagellomer-Sensilla Coeloconica-Number: (7–10)and (11–13); Antenna Flagellomer-Sensilla Coeloconica IV-X: presence; Palpus-size: wide and/or narrow opening and shallow pit; Palpus-Shape: strongly swollen; Antenna-Short sensilla trichodea, distal part segment IV to X-Number: 2 seta each) were found to be the most reliable predicting characteristics of host preference in *Culicoides* species (Table 3).

**Table 3:** Descriptive statistics on two principals components (F1 and F2 axis) based on 10, 7 and 4 morphological characteristics. Comparison with Student test between Mammalophilic/ornithophilic group and the two other groups (Ornithophilic and Mammalophilic).

Number of morphological characters	Axis	Parameters	Host preference		
			Ornithophilic (0)	Mammalophilic (M)	Mammalophilic/ ornithophilic (M/O)
10	F1	Mean ± SD	0.81 ± 0.16	−0.06 ± 0.6	−0.19 ± 0. 72
	F2		−0.34 ± 1.13	−0.02 ± 0.46	0.16 ± 0.16
7	F1		0.90 ± 0.26	−0.06 ± 0.70	−0.22 ± 0.87
	F2		0.35 ± 0.94	−0.01 ± 0.5	−0.11 ± 0.29
4	F1		1.02 ± 0.23	−0.1 ± 0.7	−0.19 ± 0.93
	F2		0.49 ± 1.43	−0.03 ± 0.38	−0.11 ± 0.30

Number of morphological characters	Axis	Parameters	**Student test between M/O and other groups**	
			Ornithophilic (0)	Mammalophilic (M)
10	F1	*P* value	*P* < 10^−4^	N.S.
	F2		N.S.	N.S.
7	F1		*P* < 10^−4^	N.S.
	F2		N.S.	N.S.
4	F1		*P* < 10^−4^	N.S.
	F2		N.S.	N.S.

**Notes.**

N.S. Not significant

## Discussion

Some aspects of the epidemiology of vector-borne diseases are linked to the host preferences and feeding behaviors of vector arthropods. This study investigates whether main sensory structures of female *Culicoides* are correlated to host species feeding preferences. Our results demonstrate that the presence of *sensilla coeloconica* and the maxillary palpus can be used to separate ornithophilic and mammalophilic or ornithophilic/mammalophilic species as previously reported on five species by Isberg, Hillbur & Ignell (2013).

Talavera et al. (2015), proposed to use only four morphological characters to predict *Culicoides* host preference based on Blackwell (2004), without statistical analysis. In the present study, seven characters are sufficient to assess host preference including the four parameters of Talavera et al. (2015). Interestingly, our results with four morphological characteristics (Fig. 2C) separate the three groups but a lot of *Culicoides* species are clustered in the same point compared to seven or 10 parameters (Figs. 2A, 2B).

Interestingly, Talavera et al. (2015) have predicted host preference for 29 *Culicoides* studied species. The current study identified 5 species as ornithophilic (*C. cataneii, C. gejgelensis*, *C. haranti, C. maritimus, C. segnis*) while there were classified as mammalophilic, 14 ornithophilic/mammalophilic species (*C. alazanicus*, *C. circumscriptus, C. festivipennis, C. griseidorsum, C. imicola, C. impunctatus, C. jumineri, C. kibunensis, C. newsteadi, C. obsoletus, C. pictipennis, C. pulicaris, C. punctatus, C. scoticus*) classified as incomplete and four species as mammalophilic (*C. brunnicans*, *C. parroti, C. puncticollis, C. shaklawensis*) while there were classified as indefinite by Talavera et al. (2015). In contrast six *Culicoides* species (*C. dewulfi*, *C. fagineus*, *C. furcillatus*, *C. lupicaris, C. poperinghensis, C. subfagineus*) are correctly attributed by the both studies.

Previous studies have suggested a relationship between the number of sensilla and host preference (Braverman & Hulley, 1979; Isberg, Hillbur & Ignell, 2013). The number of short blunt-tipped *sensilla trichodea*, *sensilla coeloconica* and s*ensilla basiconica* are significantly higher in the ornithophilic *Culicoides festivipennis* compared with the mammalophilic *C. obsoletus* and *C. scoticus* (Isberg, Hillbur & Ignell, 2013). In our study, we are unable to classify species having a higher number of *sensilla trichodea* and *sensilla coeloconica* as ornithophilic, mammalophilic or both. The ornithophilic species show a number of *sensilla coeloconica* ranging from eight to 13. The morphological sensillum types of antenna and host preference were not associated with their phylogenetic relationship (Isberg, Hillbur & Ignell, 2013) but rather with volatile organic compounds, captured by different receptors present on sensillum types (Zimmer et al., 2015).

The morphological characters of the main sensory structures of *Culicoides* and their host preferences are not linked with their breeding sites for 13, 14 and 34 of *Culicoides* studied species (Kettle & Lawson, 1952; Zimmer, Haubruge & Francis, 2014; Zimmer et al., 2014). *Culicoides* larvae develop in a wide range of wet substrates. Each species has its own requirements; therefore, larval micro-habitats are generally species-specific, even if species associations are regularly observed (Zimmer, Haubruge & Francis, 2014; Zimmer et al., 2014). The *Culicoides* species studied are recorded in the same substrates.

Finally, our study, based on 10 and seven characteristics, confirms the empirical classification of *Culicoides* into ornithophilic and mammalophilic or ornithophilic/mammalophilic, whereas, Talavera et al. (2015)’s analysis was based only on four morphological characters.

##  Supplemental Information

10.7717/peerj.3478/supp-1 Data S1 List of the 53 *Culicoides* studies species with descriptors and descriptor codes used

## Additional Information and Declarations

### Data Availability

The following information was supplied regarding data availability: The raw data has been supplied as Data S1.
